# Association Between Medicare Policy Reforms and Changes in Hospitalized Medicare Beneficiaries' Severity of Illness

**DOI:** 10.1001/jamanetworkopen.2019.3290

**Published:** 2019-05-03

**Authors:** Devraj Sukul, Geoffrey J. Hoffman, Ushapoorna Nuliyalu, Julia R. Adler-Milstein, Bill Zhang, Justin B. Dimick, Andrew M. Ryan

**Affiliations:** 1 Division of Cardiovascular Medicine, Department of Internal Medicine, University of Michigan Medical School, Ann Arbor; 2 Department of Systems, Populations and Leadership, University of Michigan School of Nursing, Ann Arbor; 3 Institute for Healthcare Policy and Innovation, Ann Arbor, Michigan; 4 Center for Healthcare Outcomes and Policy, Ann Arbor, Michigan; 5 University of California San Francisco School of Medicine, San Francisco; 6 University of Michigan School of Public Health, Ann Arbor

## Abstract

**Importance:**

The measured severity of illness of hospitalized Medicare beneficiaries has increased. Whether this change is associated with payment reforms, concentrated among hospitalizations with principal diagnoses targeted by payment reform, and reflective of true increases in severity of illness is unknown.

**Objectives:**

To assess whether the expansion of secondary diagnosis codes in January 2011 and the incentive payments for health information technology under the US Health Information Technology for Economic and Clinical Health Act were associated with changes in measured severity of illness and whether those changes are reflective of true increases in underlying patient severity.

**Design, Setting, and Participants:**

This cohort study of Medicare fee-for-service beneficiary discharges (N = 47 951 443) between January 1, 2008, and August 31, 2015, used a regression-discontinuity design to evaluate changes in measured severity of illness after the expansion of secondary diagnoses. Discharge-level linear regression model with hospital fixed effects was used to evaluate changes in measured severity of illness after hospitals’ receipt of incentives for health information technology. The change in predictive accuracy of measured severity of illness on 30-day readmissions after the implementation of both policies was evaluated. Data analysis was performed from November 1, 2018, to March 5, 2019.

**Main Outcomes and Measures:**

The primary outcome was patients’ measured severity of illness determined by the number of condition categories from secondary discharge diagnosis codes. Measured severity of illness for diagnoses commonly targeted by Medicare policies and untargeted diagnoses was assessed.

**Results:**

In total, 47 951 443 discharges at 2850 hospitals were included. In 2008, these beneficiaries included 3 882 672 women (58.5%) with a mean (SD) age of 78.5 (8.4) years. In 2014, the discharges included 3 377 137 women (57.8%) with the mean (SD) age of 78.4 (8.7) years. The Centers for Medicare & Medicaid Services expansion of secondary diagnoses was associated with a 0.348 (95% CI, 0.328-0.367; *P* < .001) change in condition categories for all diagnoses, 0.445 (95% CI, 0.419-0.470; *P* < .001) for targeted diagnoses, and 0.321 (95% CI, 0.302-0.341; *P* < .001) for untargeted diagnoses. Health information technology incentives were associated with a 0.013 (95% CI, 0.004-0.022; *P* = .005) change in condition categories for all diagnoses, 0.195 (95% CI, 0.184-0.207; *P* < .001) for targeted diagnoses, and −0.016 (95% CI, −0.025 to −0.007; *P* < .001) for untargeted diagnoses. Minimal improvements in predictive accuracy were observed.

**Conclusions and Relevance:**

Changes in Centers for Medicare & Medicaid Services policies appear to be associated with increases in measured severity of illness; these increases do not appear to reflect substantive changes in true patient severity.

## Key Points

**Question:**

Did the expansion of secondary diagnosis codes in January 2011 allow more diagnosis codes to be reported per hospitalization, and were incentive payments for health information technology associated with changes in measured severity of illness?

**Findings:**

In this cohort study of 47 951 443 discharges at 2850 hospitals, expansion of secondary diagnosis coding positions was associated with a statistically significant increase in measured severity of illness among hospitalizations for all diagnoses, diagnoses commonly targeted by incentive programs, and untargeted diagnoses. Health information technology incentives were associated with a statistically significant increase in condition categories for all diagnoses and targeted diagnoses.

**Meaning:**

Changes in Medicare policies appear to be associated with increases in measured severity of illness; related policy changes may incentivize more thorough documentation of disease burden without underlying changes in patient severity.

## Teaser

This cohort study examines US Medicare inpatient claims data from 2008 through 2015 for patients’ measured severity of illness after Medicare policy changes for the expansion of secondary diagnosis codes and payment reform.

## Introduction

A number of US health care policies have sought to improve clinical quality and reduce spending among US hospitals.^1,2,3,4^ These policies require the accurate measurement of underlying patient severity to fairly assess hospital performance. Yet the policies themselves may affect how patient severity is measured. In January 2011, the Centers for Medicare & Medicaid Services (CMS) expanded the number of secondary diagnosis coding slots, capturing up to an additional 15 diagnoses (from 9 to 24) used for risk adjustment in inpatient claims.^5^ Starting in 2011, under the Health Information Technology for Economic and Clinical Health Act,^6^ hospitals could receive incentive payments for implementing electronic health records (EHRs) and meeting specific meaningful use criteria.^7^ These incentives are associated with the rapid implementation of EHRs in health systems across the United States, with more than 95% of eligible hospitals meeting meaningful use criteria by 2016.^8^

The association of these policies with the fair and accurate assessment of patient severity is unknown. The expansion of secondary diagnosis coding slots may allow for a more accurate and nuanced assessment of patient severity. Similarly, EHR capabilities may have allowed hospitals to capture more detail about patient risk. However, hospitals faced simultaneous incentives to increase patients’ measured severity of illness with the implementation of value-based payment initiatives. For instance, the Hospital Readmissions Reduction Program (HRRP), initiated in 2010, penalized hospitals with higher-than-expected readmission rates for index hospitalizations with targeted principal diagnoses (targeted diagnoses).^2^ This program may have weakened the association between measured severity and true severity of illness among hospitalized patients by creating an incentive to increase patients’ measured severity of illness.

Using national Medicare inpatient claims from 2008 through 2015, we evaluated whether 2 CMS policies (the expansion of secondary diagnosis in January 2011 and the incentive payments for health information technology under the Health Information Technology for Economic and Clinical Health Act) were associated with changes in patients’ measured severity of illness. We then evaluated whether these policies were associated with changes in the predictive accuracy of measured severity of illness on 30-day unplanned readmission.

## Methods

### Data Source

We used 100 percent Medicare Provider Analysis and Review inpatient claims for Medicare fee-for-service beneficiaries hospitalized between January 1, 2008, and August 31, 2015, and publicly available data from CMS to ascertain if and when hospitals first attested to meeting meaningful use criteria.^9^ We obtained information on hospital size, urban or rural location, teaching status, geographic region, the proportion of inpatient days covered by Medicaid insurance, and the use of EHRs from the American Hospital Association Annual Survey Database. This study was deemed exempted from review by the University of Michigan Institutional Review Board and did not require informed consent from study participants. This study followed the Strengthening the Reporting of Observational Studies in Epidemiology (STROBE) reporting guideline.

### Study Population

The study population included all discharges from January 1, 2008, to August 1, 2015, of Medicare beneficiaries in acute care hospitals in the United States. Consistent with the CMS hospital-wide readmission measure methods,^10^ we included discharges among patients who were enrolled in Medicare fee-for-service, aged 65 to 115 years, discharged alive, and enrolled in Medicare parts A and B for at least 30 days after discharge. We excluded patients who were discharged against medical advice and those with discharges for primary psychiatric diagnoses, rehabilitation, or medical treatment of cancer.^11^ We excluded hospitals that did not attest to meeting meaningful use criteria during the study period or had fewer than 50 eligible discharges in any study year. A study flow diagram is shown in eFigure 1 in the Supplement.

### Outcomes

The primary outcome was the number of condition categories for each discharge. Condition categories comprise thousands of *International Classification of Diseases, Ninth Revision (ICD-9)* diagnosis codes. They were developed to classify *International Classification of Diseases* diagnosis codes into a broad set of diseases and are used as indicators of patient comorbidities in risk-adjustment methods.^12^ Secondary outcomes included the Medicare severity diagnosis related group (MS-DRG) weight and the Hierarchical Condition Category (HCC) community score. Condition category and HCCs were derived using publicly available software, version V2213 (CMS),^11^ and calculated using the secondary diagnoses from the discharge claim. The MS-DRG weights for each fiscal year were obtained using the respective years’ Inpatient Prospective Payment System final rule files.^13,14,15,16,17,18,19,20^

The maximum number of secondary diagnosis codes that providers were allowed to enter on a single hospital discharge claim increased from 9 to 24 in January 2011.^5^ Approximately 1.4% of discharge claims before January 2011 contained more than 9 secondary diagnosis codes. Almost all of these discharges had an accretion date in 2011, suggesting that these claims were processed by CMS when 24 secondary diagnoses were allowed. As a result, we truncated the number of secondary diagnoses to the first 9 coding positions for these discharges (eTable 1 in the Supplement). We then derived condition categories and HCCs from these secondary diagnoses. We used unplanned 30-day readmissions in our analysis of the predictive accuracy of comorbidities under different policies.

### Exposures

Discharges after January 1, 2011, were exposed to CMS policy change that increased the number of secondary diagnosis coding positions. Exposure to hospitals that received incentives for health information technologies was defined by whether the patient was discharged after the middle date of the month in which the hospital attested to meeting meaningful use criteria.

### Statistical Analysis

We performed data analysis from November 1, 2018, to March 5, 2019. We allowed the association between the policy changes and study outcomes to vary across 3 cohorts: all discharges, discharges among patients with a diagnosis that was targeted under the HRRP (acute myocardial infarction, heart failure, and pneumonia; eTable 2 in the Supplement), and discharges for untargeted diagnoses (all other conditions).

We tested the association between the expansion of secondary diagnosis coding positions and patients’ measured severity of illness using a regression-discontinuity design.^21^ The discontinuity occurred on January 1, 2011. The outcome variable was the number of condition categories and the running variable, determining exposure to the intervention, was the discharge date. Using a fourth-order polynomial, we modeled the data before and after January 2011 and included a bandwidth spanning the entire study period.^22^ Robust bias-corrected estimates of the change in measured severity of illness were obtained using a user-written command for Stata software, version 15.1 (StataCorp LLC).^23^ We also performed a falsification test^24^ to evaluate whether a discontinuity in patient age, which should not have been affected by the program, occurred at the time of the policy change. We then tested whether the association between expansion of secondary diagnosis coding positions and patients’ measured severity of illness varied by hospital characteristics, including hospital bed size and proportion of inpatient days covered by Medicare by hospital quartile.

To test whether hospital receipt of incentives for the meaningful use of EHRs was associated with an increase in measured severity of illness, we estimated a discharge-level linear regression model with hospital fixed-effects. We adjusted for time-varying patient and hospital characteristics, including patient age, sex, race/ethnicity (from the Medicare Beneficiary Summary File), and principal diagnosis based on the Healthcare Cost and Utilization Project Single-level Clinical Classifications Software^25^ as well as hospital size, geographic location (urban or rural), teaching status, hospital profit status, and proportion of inpatient days covered by Medicaid insurance. We accounted for secular trends by including discharge quarter and year dummy variables (eMethods in the Supplement). In a sensitivity analysis using similar model specifications, we assessed whether hospital EHR use was associated with changes in measured severity of illness before and after the enactment of the HRRP in April 2010. The EHR variable was missing for 18.2% of discharges. We used multiple imputation for the missing values (eMethods in the Supplement).

We evaluated whether changes in measured severity of illness after the implementation of these policies represented a more accurate assessment of patients’ true underlying severity of illness using 30-day readmission as an outcome. First, we stratified the sample into cohorts that were exposed or not exposed to the policies. To evaluate the expansion of secondary diagnosis coding positions, we grouped patients discharged after January 1, 2011, into the exposed cohort. To evaluate incentives for health information technology, we grouped patients discharged from hospitals, after the hospitals had attested to meeting meaningful use criteria, into the exposed cohort; those discharged from hospitals, before the hospitals had attested to meeting meaningful use criteria, were grouped into the not exposed cohort. For each cohort, we fit a logistic regression model adjusting for patient and hospital characteristics but not including binary indicators for individual condition categories. The outcome was a 30-day unplanned readmission.

Next, we fit the same model after adding binary indicators of individual condition categories. The difference in the C statistic between these 2 models (ie, with condition categories vs without condition categories in the exposed and unexposed cohorts) was interpreted as the incremental improvement in the predictive accuracy owing to the inclusion of patients’ measured severity of illness. The difference between the differences in C statistics for the exposed and unexposed cohorts was calculated. We interpreted this difference as the differential improvement in predictive accuracy associated with the policy changes. Using the predicted probabilities of readmission from each model, 95% CIs were bootstrapped using random sampling with replacement of all hospitals and 500 replications.

The SEs were robust to heteroscedasticity at the hospital level and CIs were constructed for 2-sided hypothesis tests. A 2-sided *P* < .05 was considered statistically significant. All analyses were conducted using Stata, version 15.1 (StataCorp LLC).

## Results

A total of 47 951 443 discharges at 2850 hospitals during the study period were included. The characteristics of discharges in 2008 (of these discharges, 3 882 672 [58.5%] were female and the mean [SD] age was 78.5 [8.4] years) and 2014 (of these discharges, 3 377 137 [57.8%] were female and the mean [SD] age was 78.4 [8.7] years) are shown in Table 1. Approximately 14% of discharges in 2008 and in 2014 were for targeted diagnoses. Between 2008 and 2015, the mean number of condition categories increased from 1.70 to 2.67 (eFigure 2A in the Supplement), the MS-DRG weight increased from 1.50 to 1.64 (eFigure 2B in the Supplement), and the HCC score increased from 1.23 to 1.69 (eFigure 2C in the Supplement). Over the same period, the mean number of condition categories increased from 2.24 to 3.44 for targeted diagnoses and from 1.61 to 2.54 for untargeted diagnoses.

**Table 1.** Baseline Characteristics of Discharges and Hospitals, 2008 and 2014

Variable	No. (%)	
	2008	2014^a^
Patient characteristics		
Unique discharges	6 639 665	5 846 612
Unique beneficiaries	4 406 070	3 986 592
Age, mean (SD), y	78.5 (8.4)	78.4 (8.7)
Female	3 882 672 (58.5)	3 377 137 (57.8)
Race/ethnicity of beneficiary		
White	5 715 234 (86.1)	4 966 121 (84.9)
Black	611 302 (9.2)	565 310 (9.7)
Hispanic	119 753 (1.8)	104 272 (1.8)
Other	193 376 (2.9)	210 909 (3.6)
No. of condition categories, mean (SD)	1.7 (1.3)	2.5 (2.0)
Hierarchical condition category score, mean (SD)	1.2 (0.8)	1.6 (1.1)
Diagnosis related group weight, mean (SD)	1.5 (1.3)	1.6 (1.4)
30-d readmission	1 062 360 (15.6)	824 708 (14.5)
Discharges with diagnoses targeted under the HRRP	910 866 (13.7)	795 550 (13.6)
Most common CCS discharges		
1 CCS - 108 (heart failure)	398 062 (6.0)	342 710 (5.9)
2 CCS - 2 (septicemia)	227 960 (3.4)	409 527 (7.0)
3 CCS - 203 (osteoarthritis)	288 574 (4.4)	322 612 (5.5)
4 CCS - 122 (pneumonia)	325 065 (4.9)	258 547 (4.4)
5 CCS - 106 (cardiac dysrhythmia)	297 711 (4.5)	245 168 (4.2)
Hospital characteristics		
Unique hospital	2850	2850
Proportion of Medicaid days	18 (12)	20 (12)
Member of Council of Teaching Hospitals	257 (9.0)	224 (7.9)
Region		
Midwest	677 (23.8)	677 (23.8)
Northeast	463 (16.2)	463 (16.2)
South	1180 (41.4)	1180 (41.4)
West	530 (18.6)	530 (18.6)
Bed size		
<200	1589 (55.8)	839 (29.4)
200-349	698 (24.5)	812 (28.5)
350-499	305 (10.7)	763 (26.8)
≥500	258 (9.1)	436 (15.3)
Hospital profit status		
For profit	560 (19.6)	618 (21.7)
Not for profit	1812 (63.6)	1788 (62.7)
Other	478 (16.8)	444 (15.6)
Hospital location		
Rural	267 (9.4)	261 (9.2)
Urban	2583 (90.6)	2589 (90.8)
Electronic health record use		
None	390 (13.7)	5 (0.2)
Partial	1251 (43.9)	510 (17.9)
Full	481 (16.9)	1534 (53.8)
Missing	728 (25.5)	801 (28.1)

Abbreviations: CCS, Clinical Classifications Software; HRRP, Hospital Readmissions Reduction Program.

^a^ 2014 was the last full year in which data were available in the data set. Data analyzed through third quarter 2015.

### Expansion of Secondary Diagnoses and Measured Severity of Illness

**Figure 1.**
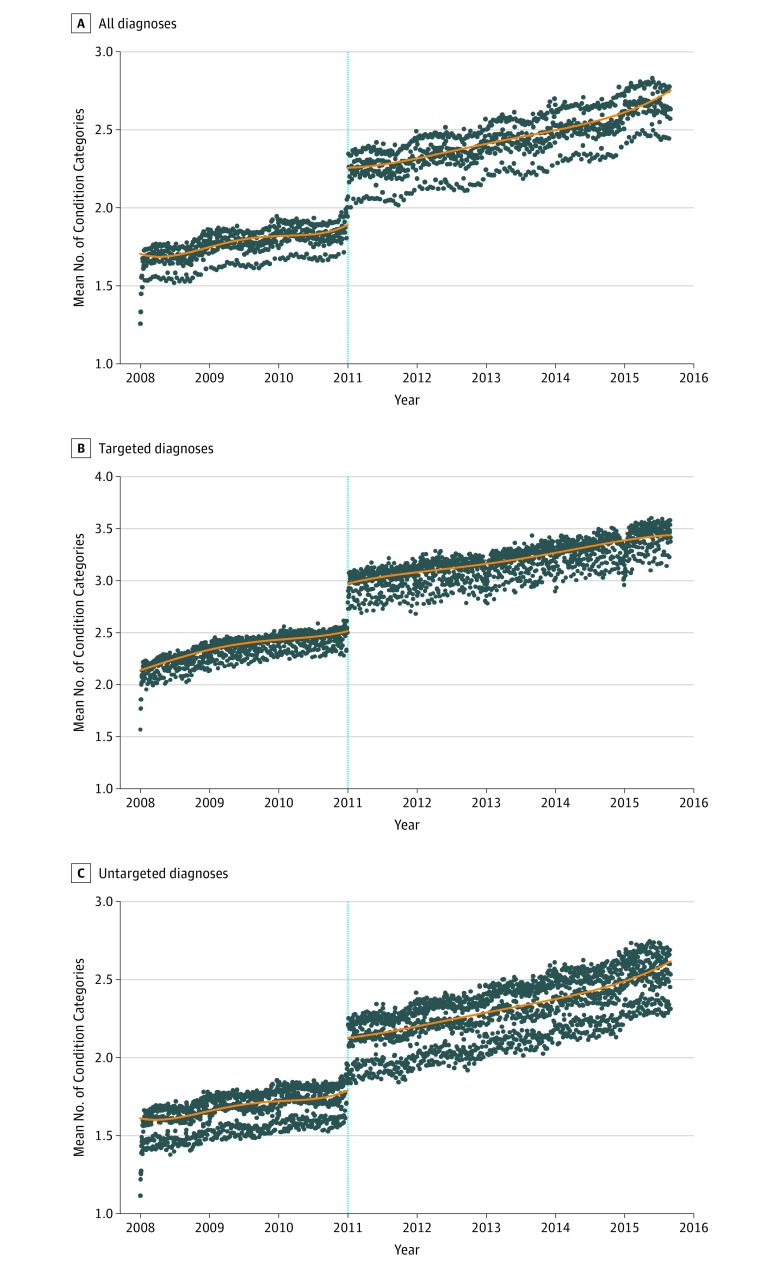
Trends in the Number of Condition Categories, 2008 to 2015 Centers for Medicare & Medicaid Services expanded the number of secondary diagnosis codes from 9 to 24 on January 1, 2011 (vertical blue line). Each point represents the mean number of condition categories derived from the secondary diagnosis codes on the discharge claim for an interval that is approximately equal to 1 day. For each panel, the blue line represents fourth-order polynomial regressions modeled with a sharp discontinuity on January 1, 2011. The robust bias-corrected estimate of the associated expansion of the number of secondary diagnosis coding positions for all diagnoses was 0.348 (95% CI, 0.328-0.367) (A); prelevel estimate of 1.89, change of 18.4%, for targeted diagnoses was 0.445 (95% CI, 0.419-0.4700 (B); prelevel estimate of 2.52, change of 17.7%, and untargeted diagnoses was 0.321 (95% CI, 0.302- 0.341; prelevel estimate of 1.79, change of 17.9%) (C).

The expansion of secondary diagnoses was associated with an estimated change of 0.348 (95% CI, 0.328-0.367; *P* < .001; change, 18.4%) in condition categories for all diagnoses, 0.445 (95% CI, 0.419-0.470; *P* < .001; change, 17.7%) for targeted diagnoses, and 0.321 (95% CI, 0.302-0.341; *P* < .001; change, 17.9%) for untargeted diagnoses (Figure 1). These findings were robust to falsification testing (eFigure 3 in the Supplement).

**Figure 2.**
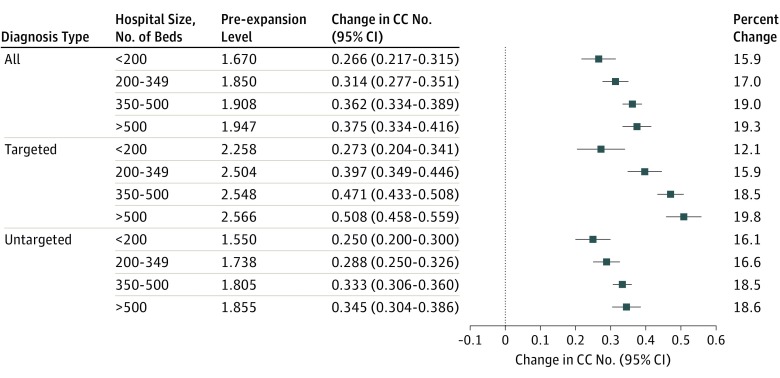
Association Between the Expansion of Secondary Diagnosis Coding Positions and Number of Condition Categories (CC), Stratified by Hospital Size The forest plot depicts the regression-discontinuity estimates of the change in the CC count after the expansion of secondary diagnosis coding positions from 9 to 24 on January 1, 2011. The prepolicy level was estimated from the fourth-order polynomial regressions used in the regression-discontinuity model at the time of the discontinuity on January 1, 2011. The percent change was calculated as the change in CC at the discontinuity from the pre-expansion level.

The association between the expansion of secondary diagnosis coding positions and measured severity of illness varied by hospital size (Figure 2). Larger hospitals experienced a greater absolute and relative change in the number of condition categories. These differences were more pronounced for targeted diagnoses compared with untargeted diagnoses. We did not see substantial heterogeneity in measured severity of illness across hospitals’ proportion of inpatient days covered by Medicare (eFigure 4 in the Supplement).

### Incentives for Health Information Technology and Severity of Illness

**Figure 3.**
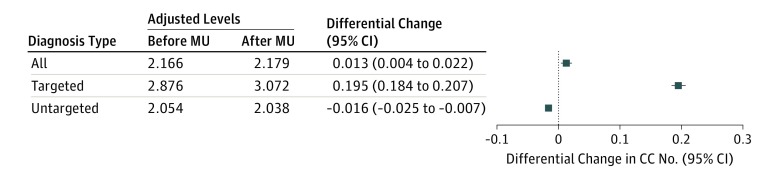
Change in the Number of Condition Categories (CC) Before and After Hospital Receipt of Health Information Technology Incentives The differential change in measured severity among all, targeted, and untargeted diagnoses is presented after controlling for patients age, sex, race/ethnicity, and principal diagnosis based on the Healthcare Cost and Utilization Project Single-Level Clinical Classifications Software as well as hospital size, geographic location (urban or rural), teaching status, and proportion of inpatient days covered by Medicaid insurance, and the quarter and year of discharge. The adjusted numbers of CCs before and after hospital attestation to meaningful use (MU) criteria are presented.

Incentives for meeting health information technology criteria were associated with a modest change in the number of condition categories for all diagnoses (0.013; 95% CI, 0.004-0.022; *P* = .005) (Figure 3; eFigure 2 and eTable 3 in the Supplement). A heterogeneous association was seen when stratified by targeted and untargeted diagnoses: incentives for health information technology were associated with a 0.195 (95% CI, 0.184-0.207; *P* < .001) change for targeted diagnoses and a −0.016 (95% CI, −0.025 to −0.007; *P* < .001) change for untargeted diagnoses. A similar pattern was noted with HCC scores (eFigure 5 in the Supplement). However, an opposite pattern was seen for MS-DRG weights with a 0.001 (95% CI, −0.003 to 0.006) change in weights for all diagnoses, a −0.059 (95% CI, −0.064 to −0.053) change for targeted diagnoses, and a 0.011 (95% CI, 0.006-0.015) change for untargeted diagnoses (eFigure 5 in the Supplement).

A sensitivity analysis evaluating changes in outcomes before and after HRRP by hospitals’ EHR use found that, as the degree of EHR use increased, the differential change in measured severity of illness increased (eFigure 6 in the Supplement). Again, the opposite pattern was seen for the outcome of MS-DRG weight.

### Predictive Accuracy of Measured Severity of Illness on 30-Day Readmission

The predictive accuracy of 30-day unplanned readmission was modest (Table 2). The differential change in the C statistic from adding condition category indicators before and after the expansion of secondary diagnoses was 0.90% (95% CI, 0.86%-0.95%) among all diagnoses, 0.95% (95% CI, 0.79%-1.1%) among targeted diagnoses, and 0.81% (95% CI, 0.76%-0.87%) among untargeted diagnoses (Table 2). The differential change in the C statistic from adding condition category indicators before and after meaningful use was 0.67% (95% CI, 0.62%-0.72%) among all diagnoses, 0.68% (95% CI, 0.53%-0.84%) among targeted diagnoses, and 0.60% (95% CI, 0.54%-0.65%) among untargeted diagnoses (Table 2).

**Table 2.** Differences in the Predictive Accuracy of Measured Severity on 30-Day Unplanned Readmission Before and After Medicare Policy Changes

Medicare Policy Change	Model C Statistic (95% CI), %			
	Without Condition Categories	With Condition Categories	Difference	Difference of Differences^a^
**Expansion of Secondary Diagnosis Coding Positions**				
All diagnoses				
Before	60.9 (60.8-6.01)	64.7 (64.6-64.7)	3.8 (3.7-3.8)	0.90 (0.86-0.95)
After	60.4 (60.3-60.5)	65.1 (65.0-65.2)	4.7 (4.6-4.7)	
Targeted diagnoses				
Before	56.2 (56.1-56.4)	60.3 (60.1-60.4)	4.0 (3.9-4.1)	0.95 (0.79-1.1)
After	56.3 (56.2-56.5)	61.3 (61.2-61.4)	5.0 (4.8-5.1)	
Untargeted diagnoses				
Before	60.9 (60.8-61.0)	64.9 (64.8-65.0)	4.0 (3.9-4.0)	0.81 (0.76-0.87)
After	60.5 (60.4-60.6)	65.3 (65.2-65.4)	4.8 (4.7-4.8)	
**Incentives for Health Information Technology**				
All diagnoses				
Before	60.7 (60.6-60.8)	64.8 (64.7-64.9)	4.1 (4.0-4.1)	0.67 (0.62-0.72)
After	60.3 (60.2-60.4)	65.1 (65.0-65.2)	4.8 (4.7-4.8)	
Targeted diagnoses				
Before	56.3 (56.2-56.4)	60.6 (60.5-60.7)	4.3 (4.2-4.4)	0.68 (0.53-0.84)
After	56.4 (56.3-56.6)	61.4 (61.3-61.6)	5.0 (4.9-5.1)	
Untargeted diagnoses				
Before	60.8 (60.7-60.9)	65.1 (64.9-65.1)	4.3 (4.2-4.3)	0.60 (0.54-0.65)
After	60.5 (60.4-60.6)	65.3 (65.2-65.4)	4.9 (4.8-4.9)	

^a^ The difference of differences is the difference between the after and before. The 95% CIs were bootstrapped from a random sample with replacement of all hospitals and 500 replications.

## Discussion

In this cohort study that evaluated changes in patients’ measured severity of illness in the context of multiple policy reforms, we report 4 principal findings. First, CMS expansion of secondary diagnosis coding positions beginning in January 2011 was associated with a large and abrupt increase in measured severity of illness. Larger hospitals experienced the greatest increase in measured severity of illness. Second, incentives for health information technology were associated with a modest increase in measured severity of illness. Third, the increases in measured severity of illness associated with incentives for health information technology were concentrated among diagnoses targeted under HRRP. Fourth, changes in measured severity of illness under these policies were associated with minimal improvements (approximately 1%) in the predictive accuracy of 30-day unplanned readmission.

To our knowledge, this study is the first to evaluate heterogeneous changes in measured severity of illness associated with CMS expansion of secondary diagnosis coding positions and hospital incentives for implementation of health information technologies. Ibrahim et al^26^ reported that increases in coded severity among targeted diagnoses were associated with a substantial proportion of the estimated reductions in readmissions after the implementation of the HRRP. Ody et al^27^ also reported that the expansion of secondary diagnosis coding positions was associated with an increase in patients’ measured severity of illness. We expand on this finding by demonstrating the heterogeneity of these associations across diagnoses that were commonly targeted and untargeted in accountability programs and across types of hospitals. Compared with untargeted diagnoses, targeted diagnoses had a greater absolute change in measured severity of illness, although the percentage changes were similar. We also found that larger hospitals had greater increases in measured severity of illness after the expansion of secondary diagnosis coding positions, particularly for targeted diagnoses. This finding suggests possible intentionality in increased coding. For instance, larger hospitals may have more available resources to facilitate increased coding and use of secondary diagnosis positions after expansion. An alternative explanation may be that larger hospitals care for sicker patients; therefore, such hospitals may have had a greater need for expanded secondary diagnosis coding positions to more completely characterize the complexity of their patients. Nonetheless, the heterogeneity of changes in measured severity of illness may have affected hospitals’ penalty status under various Medicare policies, including HRRP.

Previous research on the association of health information technologies with patients’ measured severity of illness has shown mixed results. Singh et al^28^ showed EHRs facilitated the upcoding of evaluation and management codes in a large ophthalmologic practice. It has also been speculated that EHRs may be associated with upcoding and increased payments.^29,30^ Conversely, comparing EHR adopters with controls, Adler-Milstein and Jha^31^ reported no significant difference in hospital payments or in the change of a hospital’s case-mix index, a measure closely related to MS-DRG weights. Although we found an association between health information technology and measured severity of illness captured by secondary diagnoses, we also found limited changes in MS-DRG weights associated with CMS policy changes.

The accuracy of the 30-day unplanned readmission prediction models we used was consistent with that of other published models.^32^ Yet this study is the first, to our knowledge, to evaluate the accuracy of these changes in measured severity of illness. The inclusion of condition categories into the risk-prediction models in cohorts exposed to CMS policies, compared with those unexposed, demonstrated minimal changes in the predictive capacity of the models after policy implementation. This finding, along with evidence of abrupt increases in measured severity of illness after the expansion of secondary diagnoses, suggests that the increases in measured severity of illness more likely represent changes in hospital coding practices rather than actual changes in underlying patient severity.

To fairly assess hospital performance, CMS should identify better strategies to capture patients’ true underlying severity of illness, including more intensive efforts to audit secondary diagnoses coded by hospitals.^33^ It should also consider the complex interactions among multiple contemporaneous policies, as some may be unintentionally countervailing.

### Limitations

Although other indices of patient severity are available,^34,35,36,37^ we chose the number of condition categories derived from secondary diagnoses on the discharge claim because condition categories are used in practice to estimate hospitals’ risk-adjusted performance on important outcome measures for many policies, including the HRRP.^38^ Condition categories are typically derived from 1 year’s worth of previous claims. Therefore, our derivation of condition categories from secondary diagnoses from the discharge claim alone, rather than 1 year's worth of previous claims, deviates from the traditional use. However, because we were attempting to evaluate the changes in measured severity of illness for hospitalizations, not the overall changes in measured severity of illness across the continuum of care, our outcome appropriately captures hospital coding practices. Other outcomes may yield different results, but 30-day readmission is a central focus of delivery system reform, and understanding its association with patient comorbidities is a priority for national policy.^39^

We limited our analysis to hospitals that attested to the meaningful use of EHRs during the study period and used a hospital fixed-effects design to estimate the association between health information technology adoption within hospitals and patients’ measured severity of illness. Most hospitals attested to the meaningful use of EHRs, and the small minority that did not would not be an appropriate control group as they differ in important and possibly unobservable ways. Our measurement of meaningful use also did not measure participation in the Medicaid meaningful use program. The timing of the attestation date in this analysis does not conform precisely to changes in EHR functionality. Yet these limitations would likely bias our estimates toward the null. We found a similar pattern when we specified the use of health information technologies using hospitals’ self-reported EHR use.

Although we accounted for secular trends as well as patient and hospital characteristics, it is possible that other unobserved factors may partially explain the association between policy changes and changes in measured severity of illness. However, the falsification test result from the regression-discontinuity analysis suggests that another relevant patient factor, such as age, did not meaningfully change as secondary diagnoses were expanded.

Because the details of the risk-adjustment methods used to calculate hospital performance measures in the HRRP were not released until 2012,^40^ hospitals may not have had enough information to direct changes in coding practices. However, after the passage of the Affordable Care Act in 2010, the general structure of the HRRP, including its use of risk adjustment, was clearly described.^41^ Hospitals were aware that patient risk measures would be used to determine their performance, and penalty status, in the HRRP. Moreover, if hospitals did not have enough information to direct changes in coding practices, it would have biased our findings toward the null.

## Conclusions

The study findings may be important to policymakers seeking to understand the complex interactions and consequences of multiple nationwide health care policies. The finding that increases in measured severity of illness, after hospitals met the criteria for the meaningful use of health information technologies, were greatest for diagnoses targeted under the HRRP suggests that the HRRP may have provided a strong motive for hospitals to increase patients’ measured severity of illness. The CMS policies of expanding the number of secondary diagnoses and incentivizing health information technology may have provided the mechanisms for hospitals to increase measured severity of illness.

## Notes

Supplement. **eMethods.** Details of Study Methods **eTable 1.** Frequency of Non-missing Diagnosis Codes in Each Diagnostic Coding Position Before and After Medicare Expanded the Number of Secondary Diagnosis Codes from 9 to 24 on January 1, 2011 **eTable 2.** ICD-9-CM Diagnosis Codes for Hospital Readmissions Reduction Program Targeted Diagnoses: Acute Myocardial Infarction, Heart Failure, and Pneumonia Index Admissions **eTable 3.** Baseline Characteristics of Discharges and Hospitals in 2012 Stratified by Whether the Discharging Hospital Attested to Meaningful Use Before or During 2012 **eFigure 1.** Study Flow Diagram **eFigure 2.** Adjusted Trends in the Count of Condition Categories, Diagnosis-related Group Weights, and Hierarchical Condition Category Scores Among All, Targeted, and Untargeted Diagnoses by Hospital Attestation Cohorts, Years 2011-2014 **eFigure 3.** Trends in Patient Age Between 2008-2015: Regression-discontinuity Falsification Testing **eFigure 4.** The Association Between the Expansion of Secondary Diagnosis Coding Positions and Count of Condition Categories Stratified by Hospitals’ Proportion of Inpatient Days Covered by Medicare by Quartile **eFigure 5.** Change in Diagnosis-Related Group Weights and Hierarchical Condition Category Scores Before and After Hospitals’ Receipt of Incentives for Health Information Technology **eFigure 6.** Change in Measured Severity Before and After HRRP by Hospitals’ Reported Use of Electronic Health Records
