# Supplementary material for: Association Between Medicare Policy Reforms and Changes in Hospitalized Medicare Beneficiaries' Severity of Illness
Source: JAMA Netw Open. 2019 May 3;2(5):e193290. doi: 10.1001/jamanetworkopen.2019.3290 (PMC6503517; doi:10.1001/jamanetworkopen.2019.3290)
Supplement: Supplement. — eMethods. Details of Study Methods eTable 1. Frequency of Non-missing Diagnosis Codes in Each Diagnostic Coding Position Before and After Medicare Expanded the Number of Secondary Diagnosis Codes from 9 to 24 on January 1, 2011 eTable 2. ICD-9-CM Diagnosis Codes for Hospital Readmissions Reduction Program Targeted Diagnoses: Acute Myocardial Infarction, Heart Failure, and Pneumonia Index Admissions eTable 3. Baseline Characteristics of Discharges and Hospitals in 2012 Stratified by Whether the Discharging Hospital Attested to Meaningful Use Before or During 2012 eFigure 1. Study Flow Diagram eFigure 2. Adjusted Trends in the Count of Condition Categories, Diagnosis-related Group Weights, and Hierarchical Condition Category Scores Among All, Targeted, and Untargeted Diagnoses by Hospital Attestation Cohorts, Years 2011-2014 eFigure 3. Trends in Patient Age Between 2008-2015: Regression-discontinuity Falsification Testing eFigure 4. The Association Between the Expansion of Secondary Diagnosis Coding Positions and Count of Condition Categories Stratified by Hospitals’ Proportion of Inpatient Days Covered by Medicare by Quartile eFigure 5. Change in Diagnosis-Related Group Weights and Hierarchical Condition Category Scores Before and After Hospitals’ Receipt of Incentives for Health Information Technology eFigure 6. Change in Measured Severity Before and After HRRP by Hospitals’ Reported Use of Electronic Health Records [file jamanetwopen-2-e193290-s001.pdf]

Supplementary Online Content

Sukul D, Hoffman GJ, Nuliyalu U, et al. Association between Medicare policy reforms and changes in hospitalized Medicare beneficiaries' severity of illness. *JAMA Netw Open.* 2019;2(5): e193290. doi:10.1001/jamanetworkopen.2019.3290

eMethods. Details of Study Methods
eTable 1. Frequency of Non-missing Diagnosis Codes in Each Diagnostic Coding Position Before and After Medicare Expanded the Number of Secondary Diagnosis Codes from 9 to 24 on January 1, 2011
eTable 2. ICD-9-CM Diagnosis Codes for Hospital Readmissions Reduction Program Targeted Diagnoses: Acute Myocardial Infarction, Heart Failure, and Pneumonia Index Admissions
eTable 3. Baseline Characteristics of Discharges and Hospitals in 2012 Stratified by Whether the Discharging Hospital Attested to Meaningful Use Before or During 2012
eFigure 1. Study Flow Diagram
eFigure 2. Adjusted Trends in the Count of Condition Categories, Diagnosis-related Group Weights, and Hierarchical Condition Category Scores Among All, Targeted, and Untargeted Diagnoses by Hospital Attestation Cohorts, Years 2011-2014
eFigure 3. Trends in Patient Age Between 2008-2015: Regression-discontinuity Falsification Testing
eFigure 4. The Association Between the Expansion of Secondary Diagnosis Coding Positions and Count of Condition Categories Stratified by Hospitals' Proportion of Inpatient Days Covered by Medicare by Quartile
eFigure 5. Change in Diagnosis-Related Group Weights and Hierarchical Condition Category Scores Before and After Hospitals' Receipt of Incentives for Health Information Technology
eFigure 6. Change in Measured Severity Before and After HRRP by Hospitals' Reported Use of Electronic Health Records

This supplementary material has been provided by the authors to give readers additional information about their work.

©2019 Sukul D et al. *JAMA Network Open.*

eMethods. Details of Study Methods

Model specification for evaluating the association between hospitals' receipt of incentives for health information technology and measured severity.

Our model took the following form for episode *i*, in hospital *j*, at time *t:*

$$(1)\quad Measured\ severity_{ijt} = b_0 + b_1 quarter_t + b_2 year_t + b_3 Meaningful\ Use_{jt} + b_4 X_{ijt} + b_5 Z_{jt} + b_6 U_j + e_{ijt}$$

Where quarter and year are dummy variables for the calendar quarter and year of discharge, *Meaningful Use* indicates hospital receipt of incentives for the meaningful use of health information technologies, *X* is a vector of time-varying patient characteristics (age at the time of discharge, principal diagnosis according to the Healthcare Utilization Project single-level clinical classifications software [HCUP CCS], race, gender), *Z* is a vector of time-varying hospital characteristics (bed size, geographic location (urban/rural), teaching status, hospital profit status, and the proportion of inpatient days covered by Medicaid insurance), and *U* is a vector of hospital fixed-effects. The association between *Meaningful Use* and *Measured severity* is captured by b_3. Of note, the principal diagnosis according to the HCUP CCS, was excluded from models where the outcome was medical severity diagnosis-related group (MS-DRG) weight as the MS-DRG is based on the principal discharge diagnosis.

We allowed the effect of meaningful use to vary according to whether the discharge diagnosis was targeted or untargeted under the Hospital Readmissions Reduction Program (HRRP) by adding a binary variable, *HRRP DX*, indicating whether the discharge diagnosis was targeted or not, and included this as a main effect and interaction with *Meaningful Use* as follows:

$$(2)\quad Measured\ severity_{ijt} = b_0 + b_1 quarter_t + b_2 year_t + b_3 Meaningful\ Use_{jt} + b_4 HRRP\ DX_{ijt} + b_5 (HRRP\ DX_{ijt} * Meaninful\ Use_{jt}) + b_6 X_{ijt} + b_7 Z_{jt} + b_8 U_j + e_{ijt}$$

Multiple imputation accounting for the missingness of the electronic health record use variable.

The electronic health record (EHR) variable obtained from the American Hospital Association Annual Survey Database was missing from 18.6% of hospitalizations. Therefore, in sensitivity analyses using this variable, we performed multiple imputation to impute these missing values at the episode-level. Given that the EHR variable consists of three categories - no EHR, partial EHR, full EHR - we used multinomial logistic regression, including all the covariates and outcomes in the primary hospital fixed-effects model (Equation 1), to impute these values. Then, using 8 multiply imputed datasets, our model took the following form for hospitalization *i*, hospital *j*, at time *t*.

$$(3)\quad Measured\ severity_{ijt} = b_0 + b_1 quarter_t + b_2 year_t + b_3 EHR_{ijt} + b_4 (EHR_{ijt} * Post\ HRRP_t) + b_5 X_{ijt} + b_6 Z_{jt} + b_7 U_j + e_{ijt}$$

Where *EHR* is a categorical variable indicating hospitals' self-reported use of EHRs (none, partial, full), and *Post-HRRP* indicates discharges occurring after hospitals were exposed to the HRRP occurring after April 1, 2010. All other variables are as defined for equation 1. Of note, *Post HRRP* was not included as a main effect as it is collinear with the time variables. We then estimated marginal effects of *EHR* use before and after the HRRP on measured severity.

We allowed the effect of EHR use before and after HRRP on measured severity to vary according to whether the discharge diagnosis was targeted or untargeted under the HRRP by adding a binary variable, *HRRP DX*, indicating whether the discharge diagnosis was targeted or untargeted. This variable was included as a main effect and one- and two-way interactions with *EHR* and *Post HRRP* as follows:

$$(4)\quad Measured\ severity_{ijt} = b_0 + b_1 quarter_t + b_2 year_t + b_3 EHR_{ijt} + b_4 HRRP\ DX_{ijt} + b_5 \left(EHR_{ijt} * HRRP\ DX_{ijt}\right) + b_6 \left(EHR_{ijt} * Post\ HRRP_t\right) + b_7 \left(HRRP\ DX_{ijt} * Post\ HRRP_t\right) + b_8 \left(EHR_{ijt} * HRRP\ DX_{ijt} * Post\ HRRP_t\right) + b_9 X_{ijt} + b_{10} Z_{jt} + b_{11} U_j + e_{ijt}$$

We then estimated the marginal effects of *EHR* use before and after the HRRP on measured severity among targeted and untargeted diagnoses.

©2019 Sukul D et al. *JAMA Network Open.*

eTable 1. Frequency of Non-missing Diagnosis Codes in Each Diagnostic Coding Position Before and After Medicare Expanded the Number of Secondary Diagnosis Codes from 9 to 24 on January 1, 2011

Diagnostic position	Total (N=47, 951, 443)	Before 1/1/2011 (N=19, 678, 623)	After 1/1/2011 (N=28, 272, 820)
Diagnosis 1	100.00%	100.00%	100.00%
Diagnosis 2	99.72%	99.67%	99.75%
Diagnosis 3	98.97%	98.75%	99.13%
Diagnosis 4	97.44%	96.85%	97.85%
Diagnosis 5	94.85%	93.67%	95.68%
Diagnosis 6	91.11%	89.14%	92.48%
Diagnosis 7	86.29%	83.41%	88.29%
Diagnosis 8	80.57%	76.76%	83.22%
Diagnosis 9	74.17%	69.44%	77.46%
Diagnosis 10	43.35%	8.87%	70.50%
Diagnosis 11	38.16%	0.00%	64.16%
Diagnosis 12	34.34%	0.00%	57.75%
Diagnosis 13	30.58%	0.00%	51.44%
Diagnosis 14	26.71%	0.00%	44.94%
Diagnosis 15	23.34%	0.00%	39.28%
Diagnosis 16	19.87%	0.00%	33.44%
Diagnosis 17	16.97%	0.00%	28.56%
Diagnosis 18	14.41%	0.00%	24.27%
Diagnosis 19	10.57%	0.00%	17.83%
Diagnosis 20	8.91%	0.00%	15.03%
Diagnosis 21	7.45%	0.00%	12.57%
Diagnosis 22	6.03%	0.00%	10.19%
Diagnosis 23	5.02%	0.00%	8.47%
Diagnosis 24	4.07%	0.00%	6.88%
Diagnosis 25	3.05%	0.00%	5.15%

©2019 Sukul D et al. *JAMA Network Open.*

eTable 2. ICD-9-CM Diagnosis Codes for Hospital Readmissions Reduction Program Targeted Diagnoses: Acute Myocardial Infarction, Heart Failure, and Pneumonia Index Admissions

1) Acute myocardial infarction (AMI)

ICD-9-CM Codes	Description
410.00	AMI (anterolateral wall) - episode of care unspecified
410.01	AMI (anterolateral wall) - initial episode of care
410.10	AMI (other anterior wall) - episode of care unspecified
410.11	AMI (other anterior wall) - initial episode of care
410.20	AMI (inferolateral wall) - episode of care unspecified
410.21	AMI (inferolateral wall) - initial episode of care
410.30	AMI (inferoposterior wall) - episode of care unspecified
410.31	AMI (inferoposterior wall) - initial episode of care
410.40	AMI (other inferior wall) - episode of care unspecified
410.41	AMI (other inferior wall) - initial episode of care
410.50	AMI (other lateral wall) - episode of care unspecified
410.51	AMI (other lateral wall) - initial episode of care
410.60	AMI (true posterior wall) - episode of care unspecified
410.61	AMI (true posterior wall) - initial episode of care
410.70	AMI (subendocardial) - episode of care unspecified
410.71	AMI (subendocardial) - initial episode of care
410.80	AMI (other specified site) - episode of care unspecified
410.81	AMI (other specified site) - initial episode of care
410.90	AMI (unspecified site) - episode of care unspecified
410.91	AMI (unspecified site) - initial episode of care

©2019 Sukul D et al. *JAMA Network Open.*

2) Congestive heart failure (CHF)

ICD-9-CM Codes	Description
402.01	Malignant hypertensive heart disease with CHF
402.11	Benign hypertensive heart disease with CHF
402.91	Hypertensive heart disease with CHF
404.01	Malignant hypertensive heart and renal disease with CHF
404.03	Malignant hypertensive heart and renal disease with CHF & renal failure
404.11	Benign hypertensive heart disease and renal disease with CHF
404.13	Benign hypertensive heart disease and renal disease with CHF & renal failure
404.91	Unspecified hypertensive heart and renal disease with CHF
404.93	Hypertension and non-specified heart and renal disease with CHF & renal failure
428.0	Congestive heart failure, unspecified
428.1	Left heart failure
428.2	Systolic heart failure, unspecified
428.21	Systolic heart failure, acute
428.22	Systolic heart failure, chronic
428.23	Systolic heart failure, acute or chronic
428.3	Diastolic heart failure, unspecified
428.31	Diastolic heart failure, acute
428.32	Diastolic heart failure, chronic
428.33	Diastolic heart failure, acute or chronic
428.4	Combined systolic and diastolic heart failure, unspecified
428.41	Combined systolic and diastolic heart failure, acute
428.42	Combined systolic and diastolic heart failure, chronic
428.43	Combined systolic and diastolic heart failure, acute or chronic
428.9	Heart failure, unspecified

©2019 Sukul D et al. *JAMA Network Open.*

3) Pneumonia

ICD-9-CM Codes	Description
480.0	Pneumonia due to adenovirus
480.1	Pneumonia due to respiratory syncytial virus
480.2	Pneumonia due to parainfluenza virus
480.3	Pneumonia due to SARS-associated coronavirus
480.8	Viral pneumonia: pneumonia due to other virus not elsewhere classified
480.9	Viral pneumonia unspecified
481.0	Pneumococcal pneumonia (streptococcus pneumoniae pneumonia)
482.0	Pneumonia due to Klebsiella pneumoniae
482.1	Pneumonia due to Pseudomonas
482.2	Pneumonia due to Haemophilus influenzae (H. influenzae)
482.3	Pneumonia due to streptococcus unspecified
482.31	Pneumonia due to streptococcus group a
482.32	Pneumonia due to streptococcus group b
482.39	Pneumonia due to other streptococcus
482.4	Pneumonia due to staphylococcus unspecified
482.41	Pneumonia due to Staphylcoccus aureus
482.42	Methicillin resistant pneumonia due to Staphylococcus aureus
482.49	Other staphylococcus pneumonia
482.81	Pneumonia due to anaerobes
482.82	Pneumonia due to Escherichia coli (E. coli)
482.83	Pneumonia due to other gram-negative bacteria
482.84	Pneumonia due to Legionnaires' disease
482.89	Pneumonia due to other specified bacteria
482.9	Bacterial pneumonia unspecified
483.0	Pneumonia due to Mycoplasma pneumoniae
483.1	Pneumonia due to chlamydia
483.8	Pneumonia due to other specified organism
485.0	Bronchopneumonia organism unspecified
486.0	Pneumonia organism unspecified
487.0	Influenza with pneumonia
488.11	Influenza due to identified novel H1N1 influenza virus with pneumonia

©2019 Sukul D et al. *JAMA Network Open.*

eTable 3. Baseline Characteristics of Discharges and Hospitals in 2012 Stratified by Whether the Discharging Hospital Attested to Meaningful Use Before or During 2012

Characteristics	**Non-MU**	**MU**
**Patient characteristics**		
Unique discharges	3,594,570	2,399,915
Unique beneficiaries	2560843	1756737
Age	78.6 (8.6)	78.6 (8.6)
Female sex	2,104,861 (58.6%)	1, 402, 462 (58.4%)
Race of Beneficiary		
White	3,054,423 (85.0%)	2,053,045 (85.5%)
Black	344,729 (9.6%)	229,238 (9.6%)
Hispanic	70,701 (2.0%)	43,876 (1.8%)
Other	124,717 (3.5%)	73,756 (3.1%)
No. of condition categories, mean (SD)	2.3 (1.9)	2.4 (2.0)
Hierarchical condition category score, mean (SD)	1.5 (1.0)	1.5 (1.1)
Diagnosis-related group weight, mean (SD)	1.6 (1.3)	1.6 (1.3)
30-day readmission, n/N (%)	531,789 (14.8%)	361,257 (15.1%)
Discharges with diagnoses targeted under the HRRP	490,722 (13.7%)	320,373 (13.3%)
Most the most common CCS discharges		
#1 CCS - 108 (Heart failure	202, 596(5.6%)	135,345(5.6)
#2CCS - 2 (Septicemia)	186,540(5.2)	128,296(5.4)
#3CCS - 203 (Osteoarthritis)	178,204(5.0)	114,371(4.8)
#4 CCS - 122 (Pneumonia)	178,030(5.0)	109,865(4.6)
#5 CCS - 106 (Cardiac dysrhythmias)	165,226(4.6)	109,554(4.6)
**Hospital characteristics**		
Unique hospitals	1,113	1,737
Proportion of Medicaid days	18% (10%)	18% (10%)
Member of Council of Teaching Hospitals	84 (7.5%)	166 (9.6%)
Region		
Midwest	239 (21.5%)	438 (25.2%)
Northeast	146 (13.1%)	317 (18.2%)
South	482 (43.3%)	698 (40.2%)
West	246 (22.1%)	284 (16.4%)
Bed Size		
<200	475 (42.7%)	676 (38.9%)
200-349	303 (27.2%)	443 (25.5%)
350-499	205 (18.4%)	382 (22.0%)
>=500	130 (11.7%)	236 (13.6%)
Hospital profit status		

©2019 Sukul D et al. *JAMA Network Open.*

For profit	232 (20.8%)	351 (20.2%)
Not-for profit	693 (62.3%)	1,106 (63.7%)
Other	188 (16.9%)	280 (16.1%)
Electronic Health Record Use		
None	96 (8.6%)	88 (5.1%)
Partial	469 (42.1%)	556 (32.0%)
Full	252 (22.6%)	646 (37.2%)
Missing	296 (26.6%)	447 (25.7%)

* *30-day unplanned readmissions were calculated after removing patients who died within 30 days after discharge.*
Data presented as mean (standard deviation) or N (%) where appropriate.
Abbreviations: CCS = Clinical classifications software

©2019 Sukul D et al. *JAMA Network Open.*

eFigure 1. Study Flow Diagram

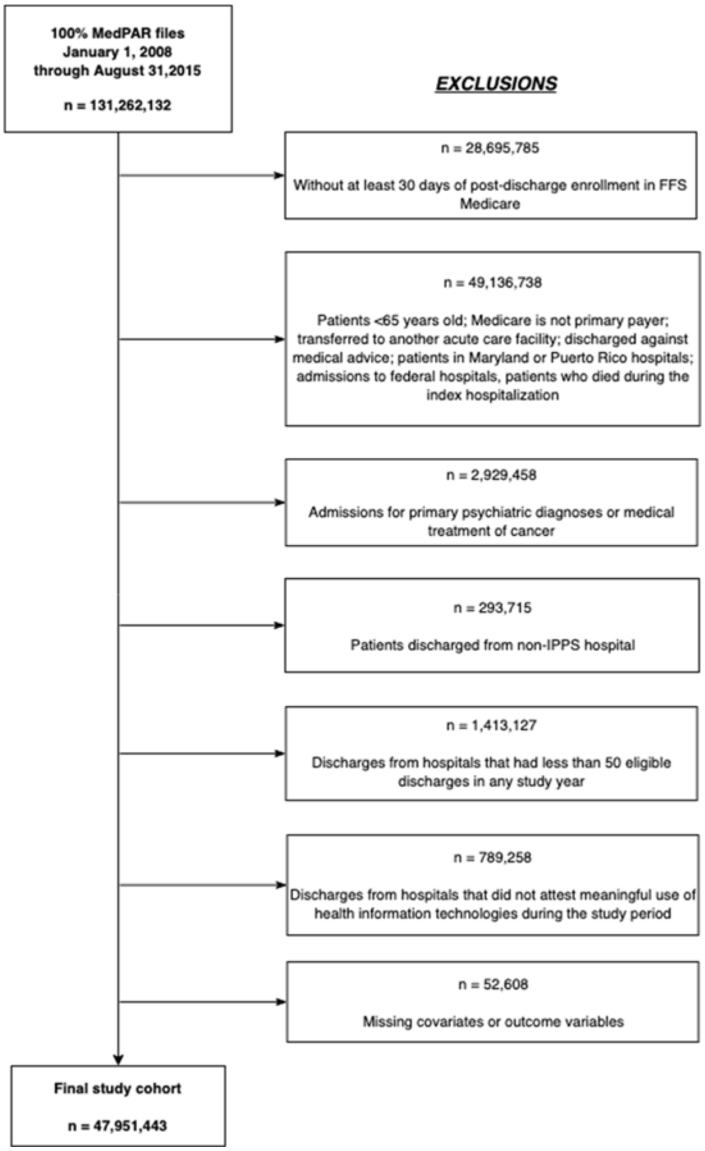

©2019 Sukul D et al. *JAMA Network Open.*

eFigure 2. Adjusted Trends in the Count of Condition Categories, Diagnosis-related Group Weights, and Hierarchical Condition Category Scores Among All, Targeted, and Untargeted Diagnoses by Hospital Attestation Cohorts, Years 2011-2014

A. Count of condition categories

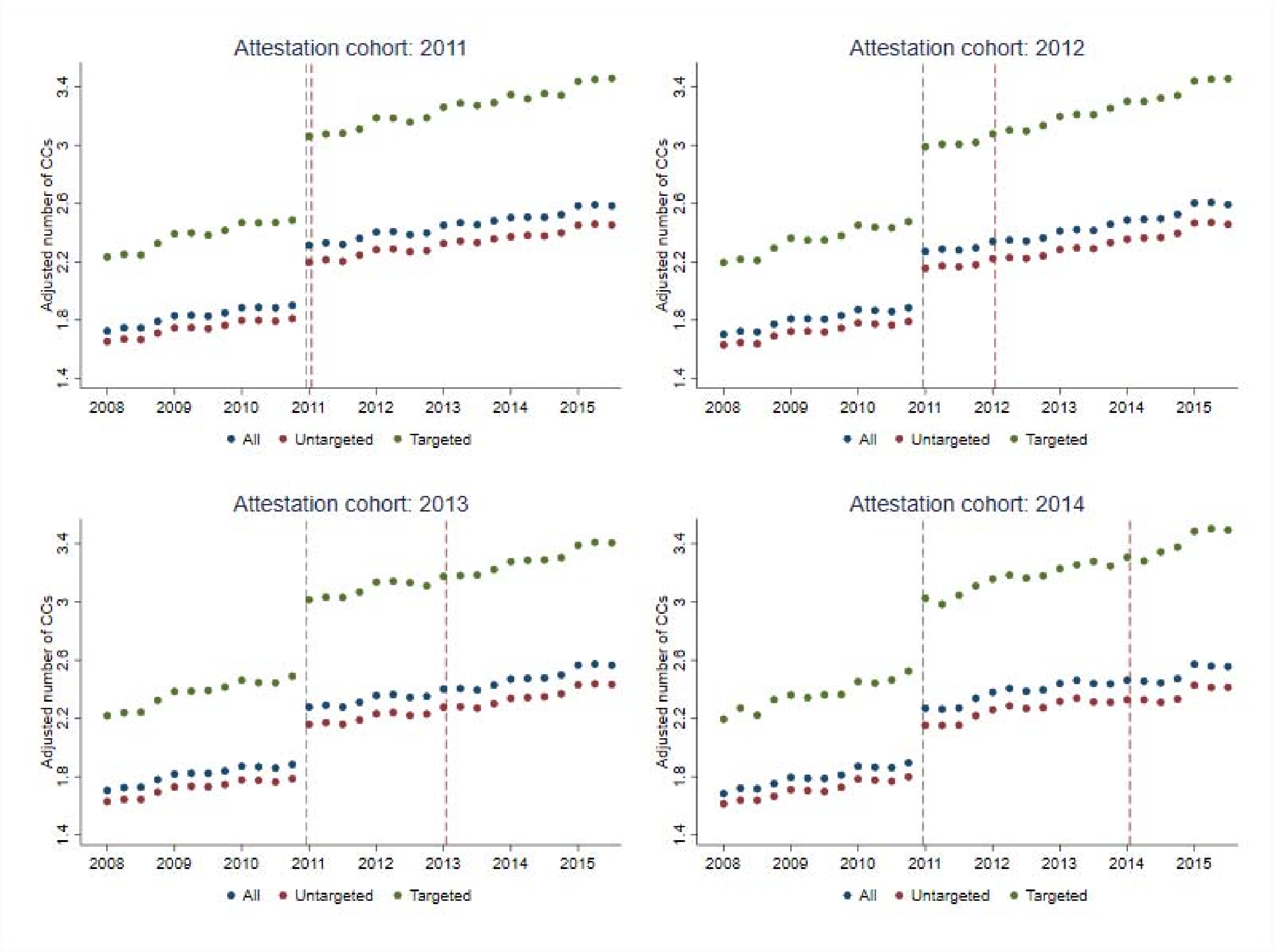

©2019 Sukul D et al. *JAMA Network Open.*

B. Diagnosis-related group weights

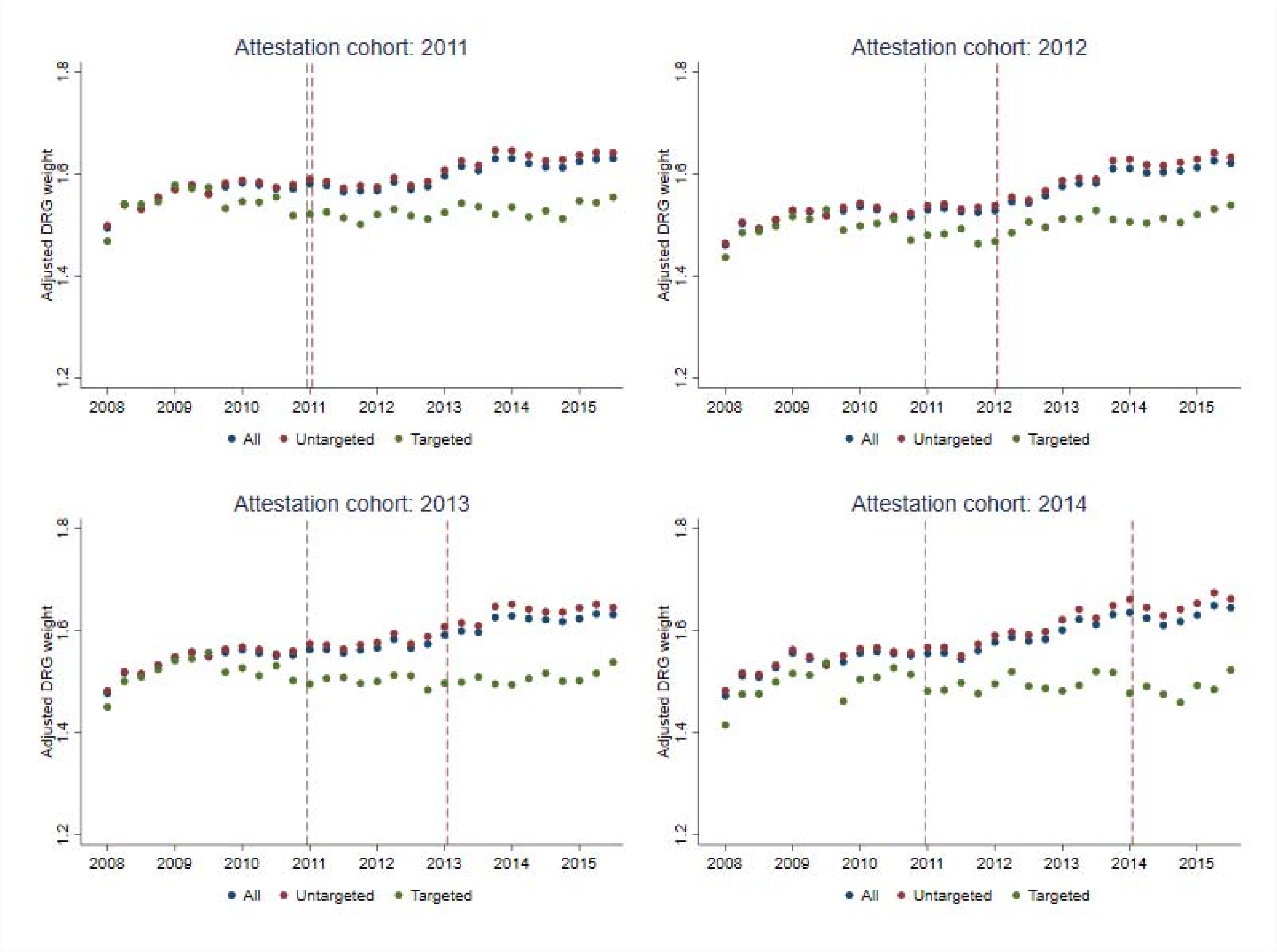

©2019 Sukul D et al. *JAMA Network Open.*

C. Hierarchical condition category score

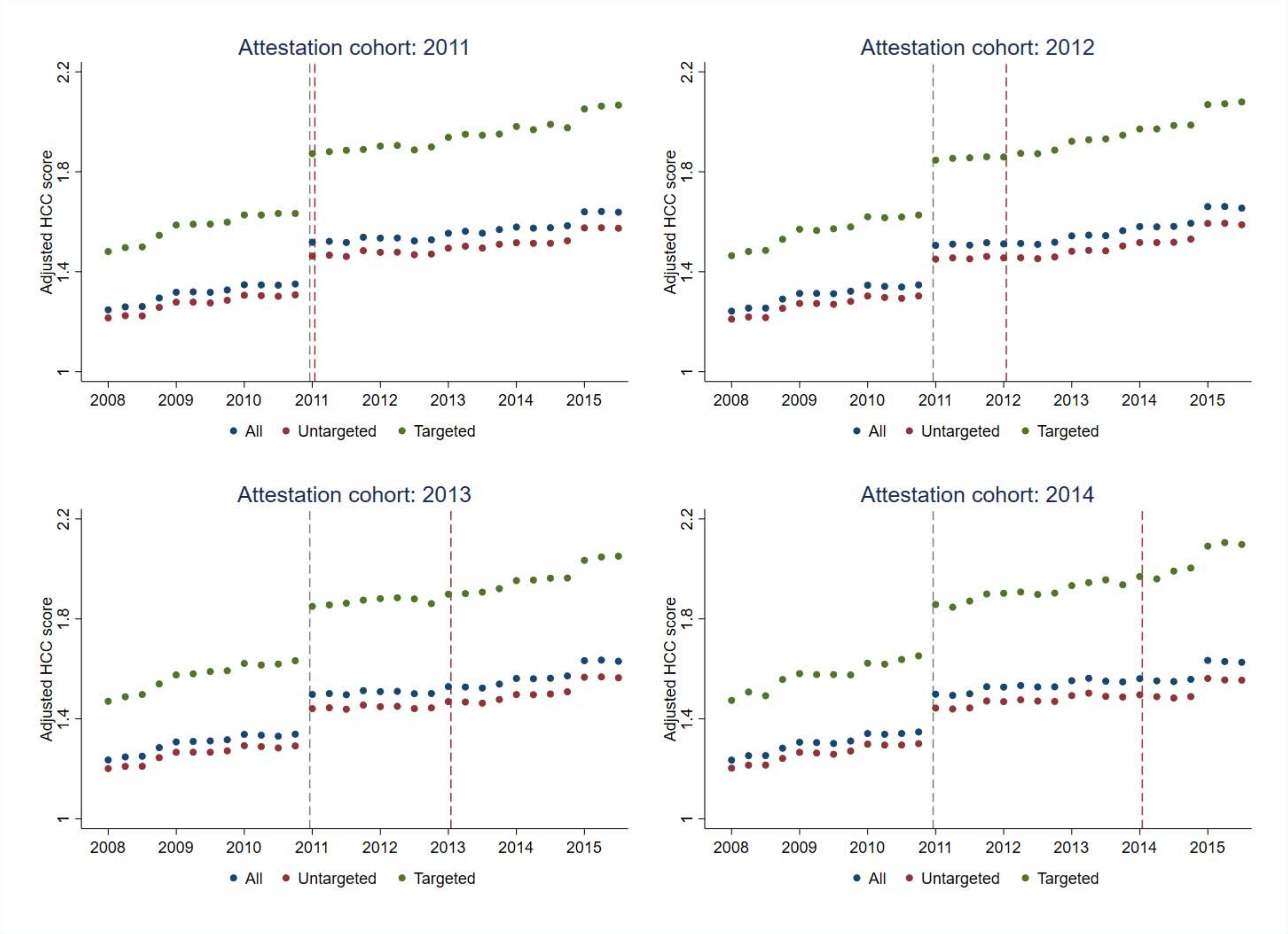

Abbreviations: CC = condition category, DRG = diagnosis-related group, HCC = hierarchical condition category score.

Notes: The red dashed line indicates the year in which the cohort of hospitals attested to meaningful use. The dashed gray line indicates when Medicare expanded the number of secondary diagnoses from 9 to 24 on January 1, 2011. Each point represents the adjusted level of the outcome after controlling for age, sex, race, the patient's principal diagnosis based on the Healthcare Cost and Utilization Project Single-level Clinical Classifications Software (except for the outcome of DRG weight), hospital size, geographic location (urban/rural), teaching status, the proportion of inpatient days covered by Medicaid insurance, and the quarter and year of discharge. The adjusted counts of CCs before and after hospital attestation to meaningful use criteria are presented.

©2019 Sukul D et al. *JAMA Network Open.*

eFigure 3. Trends in Patient Age Between 2008-2015: Regression-discontinuity Falsification Testing

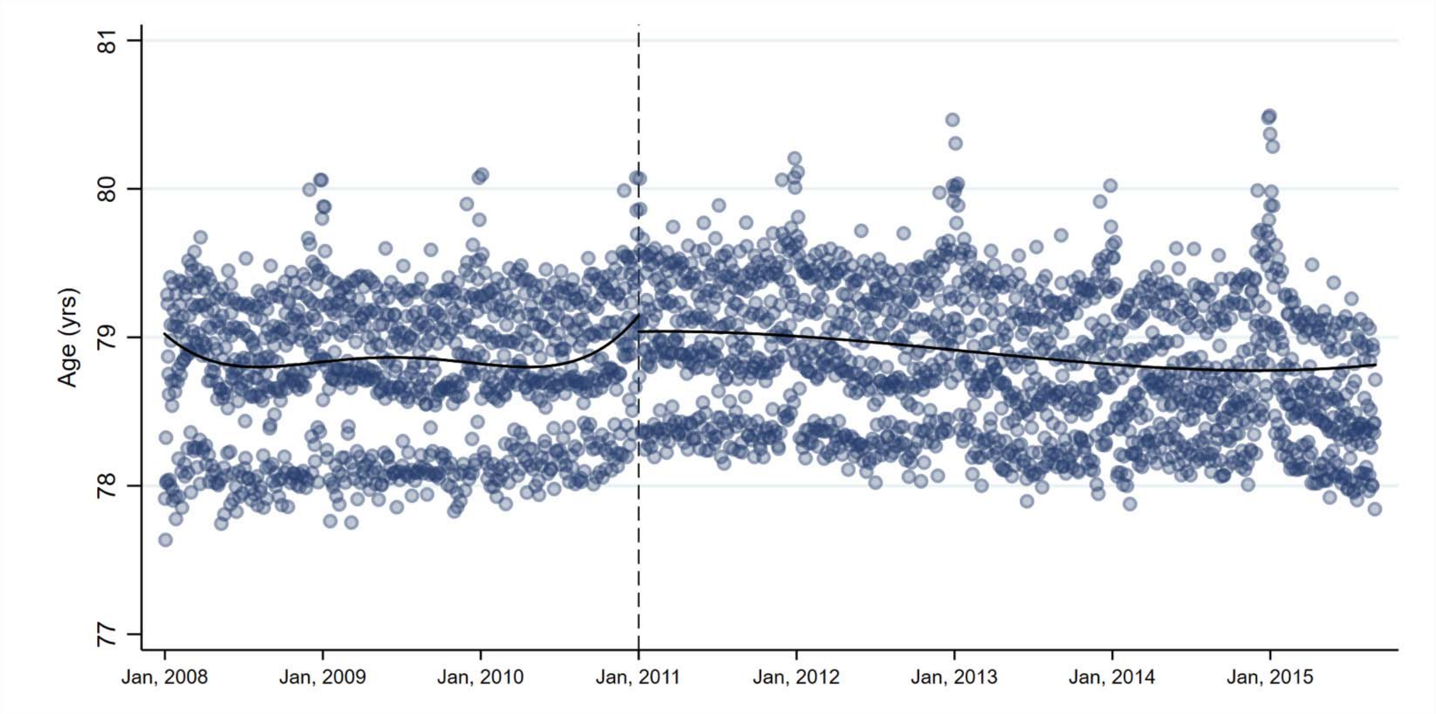

Notes: January 1, 2011 (vertical dashed line) was when Medicare expanded the number of secondary diagnosis codes from 9 to 24. Each point represents the mean age at the time of discharge for an interval that is approximately equal to one day. As performed in the primary analysis, the black lines represent 4th order polynomial regression modeled with a sharp discontinuity at January 1, 2011. The robust bias-corrected estimate of the effect of expanding the number of secondary diagnosis codes on age was -0.187 (95% confidence interval: -0.285, -0.088).

©2019 Sukul D et al. *JAMA Network Open.*

eFigure 4. The Association Between the Expansion of Secondary Diagnosis Coding Positions and Count of Condition Categories Stratified by Hospitals' Proportion of Inpatient Days Covered by Medicare by Quartile

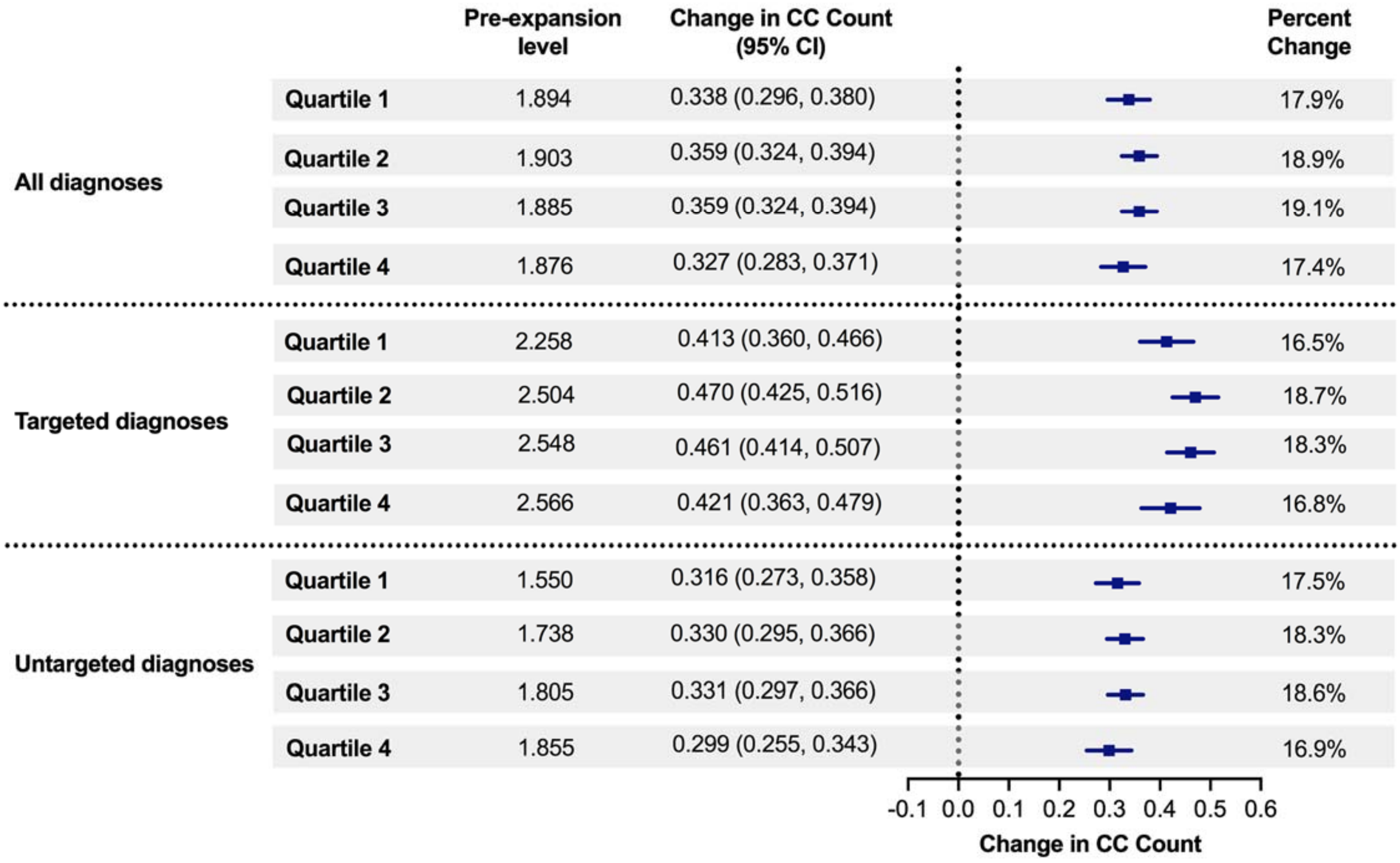

Abbreviations: CC=condition category; CI=confidence interval.

Notes: The forest plot depicts the regression-discontinuity estimates of the change in the count of condition categories after the expansion of secondary diagnosis coding positions from 9 to 24 on January 1, 2011. The pre-policy level is estimated from the 4th order polynomial regressions used in the regression-discontinuity model at the time of the discontinuity on January 1, 2011. The percent change is calculated as the change in CCs at the discontinuity relative to the pre-expansion level.

©2019 Sukul D et al. *JAMA Network Open.*

eFigure 5. Change in Diagnosis-Related Group Weights and Hierarchical Condition Category Scores Before and After Hospitals' Receipt of Incentives for Health Information Technology

A. Diagnosis-related group weights

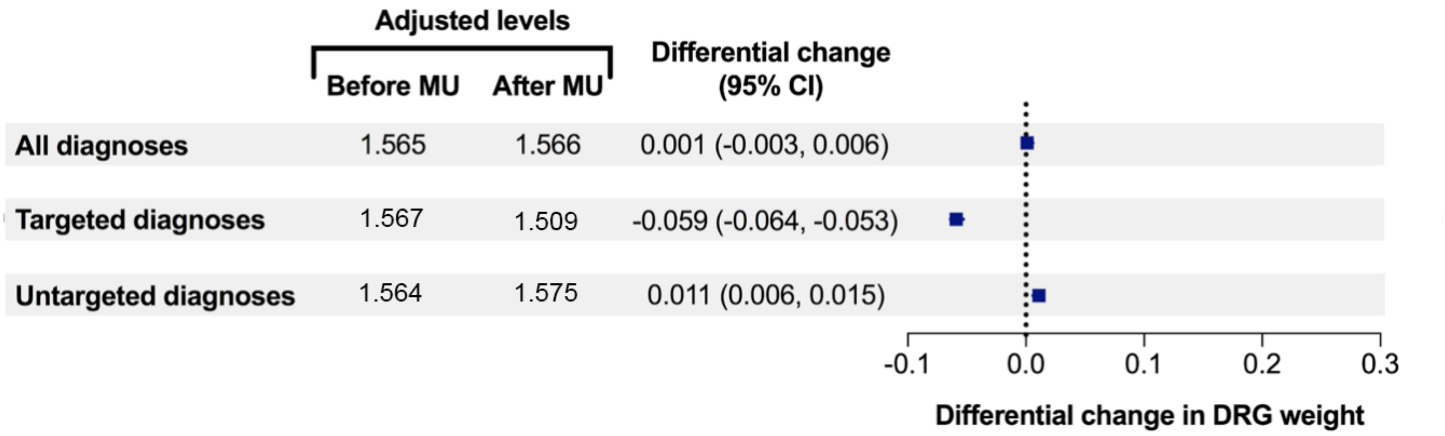

B. Hierarchical Condition Category scores

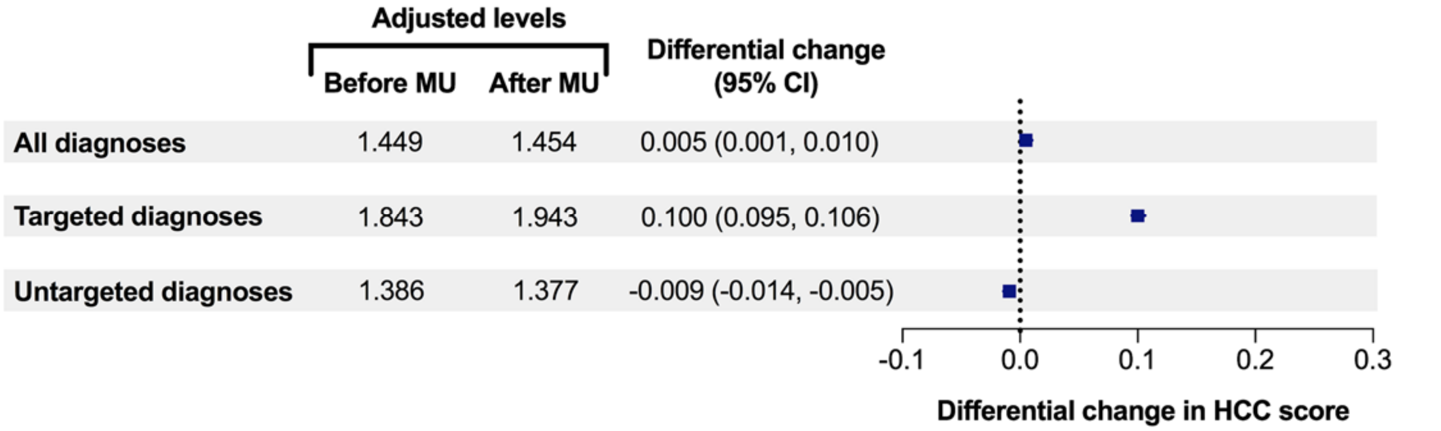

Abbreviations: DRG=diagnosis-related group, HCC=Hierarchical Condition Category, MU=Meaningful Use.
Notes: The differential change in diagnosis-related group weights and hierarchical condition category scores among all, targeted, and untargeted diagnoses is presented after controlling for age, sex, race, the patient's principal diagnosis based on the Healthcare Cost and Utilization Project Single-level Clinical Classifications Software, hospital size, geographic location (urban/rural), teaching status, the proportion of inpatient days covered by Medicaid insurance, and the quarter and year of discharge. The adjusted counts of CCs before and after hospital attestation to meaningful use criteria are presented.

©2019 Sukul D et al. *JAMA Network Open.*

eFigure 6. Change in Measured Severity Before and After HRRP by Hospitals' Reported Use of Electronic Health Records

A. Count of condition categories

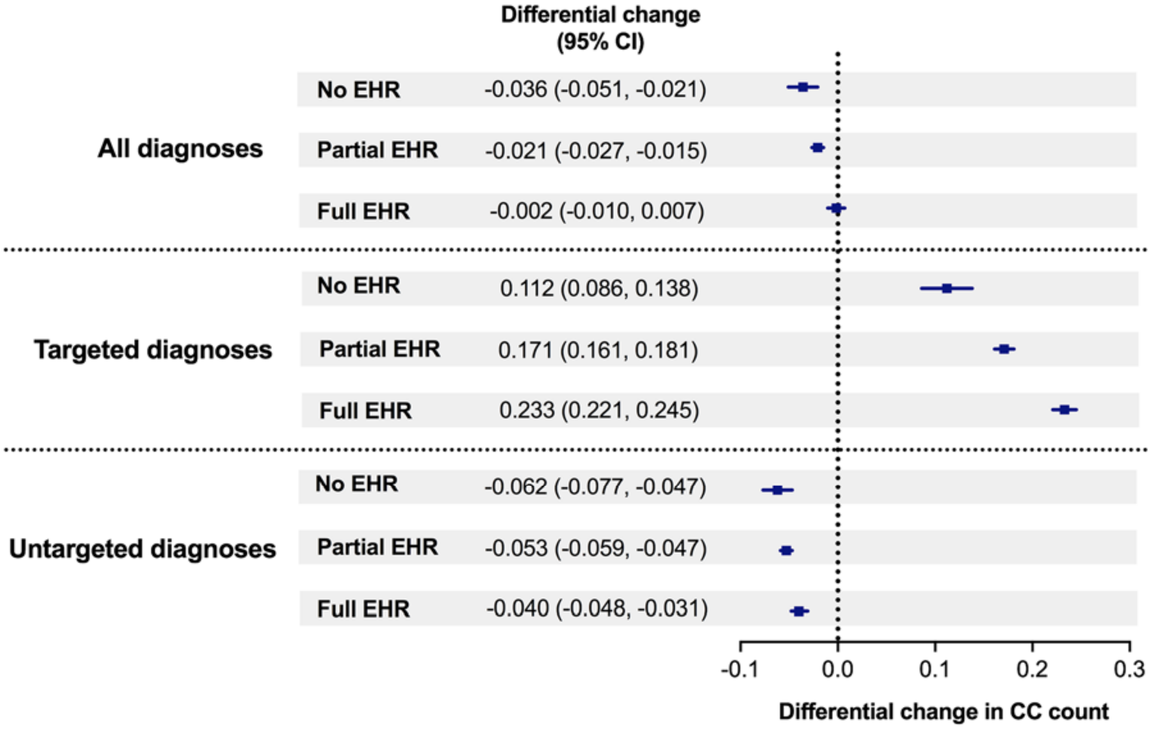

B. Diagnosis-related group weight

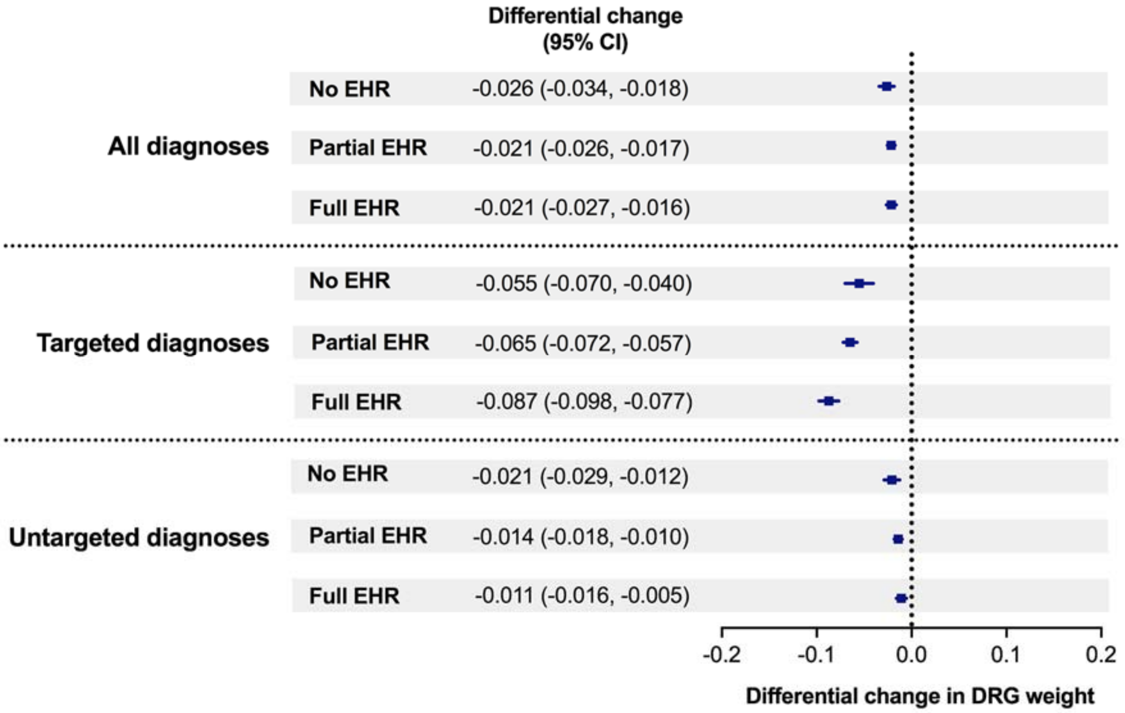

©2019 Sukul D et al. *JAMA Network Open.*

C. Hierarchical condition category score

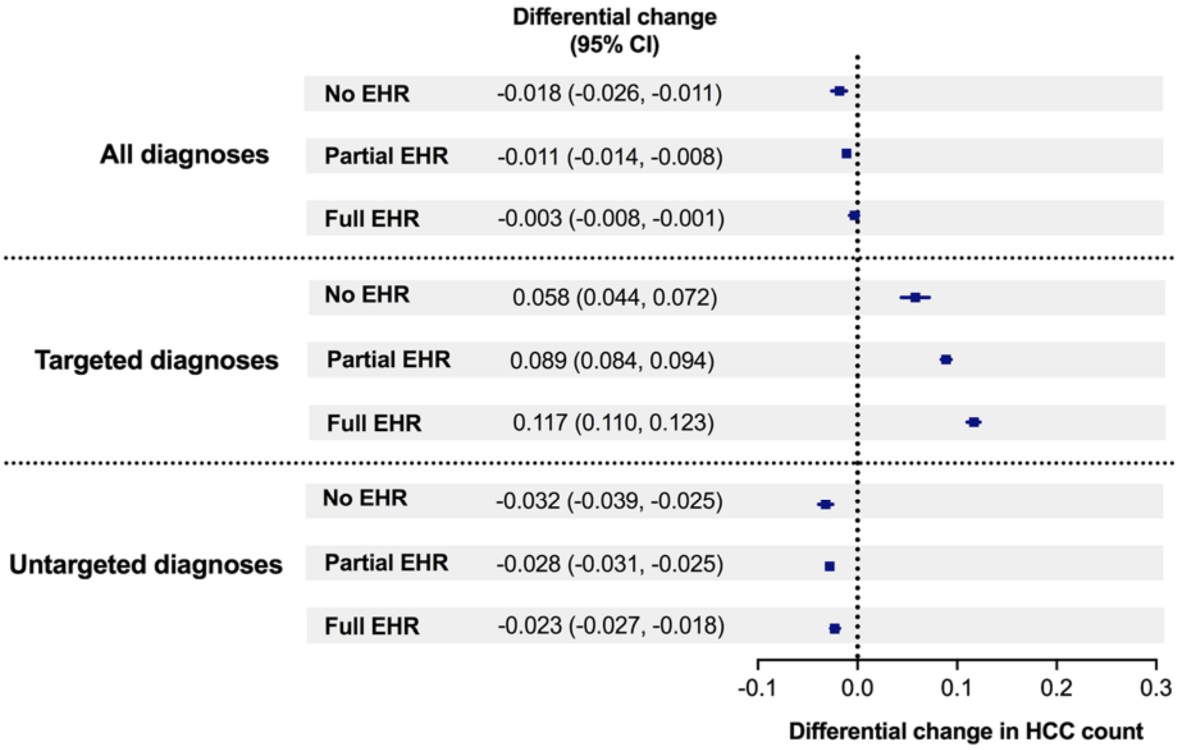

Abbreviations: CC=condition category, DRG=diagnosis-related group, EHR=electronic health record, HCC=hierarchical condition category.

Notes: The differential change in primary and secondary outcomes among all, targeted, and untargeted diagnoses is presented after controlling for age, sex, race, the patient's principal diagnosis based on the Healthcare Cost and Utilization Project Single-level Clinical Classifications Software (CCS), hospital size, geographic location (urban/rural), teaching status, the proportion of inpatient days covered by Medicaid insurance, and the quarter and year of discharge. Multiple imputation was used to account for the missingness of the hospital's electronic health record use variable.

©2019 Sukul D et al. *JAMA Network Open.*
